# Optimization and validation of the EconomicClusters model for facilitating global health disparities research: Examples from Cameroon and Ghana

**DOI:** 10.1371/journal.pone.0217197

**Published:** 2019-05-23

**Authors:** Lauren Eyler, Alan Hubbard, Catherine Juillard

**Affiliations:** 1 Department of Surgery, Center for Global Surgical Studies, University of California San Francisco, San Francisco, California, United States of America; 2 Division of Biostatistics, School of Public Health, University of California Berkeley, Berkeley, California, United States of America; 3 Department of Surgery, University of California Los Angeles, Los Angeles, California, United States of America; University of Sheffield, UNITED KINGDOM

## Abstract

Health disparities research in low- and middle-income countries (LMICs) is hampered by the difficulty of measuring economic status in low-resource settings. We previously developed the EconomicClusters k-medoids clustering-based algorithm for defining population-specific economic models based on few Demographic and Health Surveys (DHS) assets. The algorithm previously defined a twenty-group economic model for Cameroon. The aims of this study are to optimize the functionality of our EconomicClusters algorithm and app based on collaborator feedback from early use of this twenty-group economic model, to test the validity of the model as a metric of economic status, and to assess the utility of the model in another LMIC context. We condense the twenty Cameroonian economic groups into fewer, ordinally-ranked, groups using agglomerative hierarchical clustering based on mean cluster child height-for-age Z-score (HAZ), women’s literacy score, and proportion of children who are deceased. We develop an EconomicClusters model for Ghana consisting of five economic groups and rank these groups based on the same three variables. The proportion of variance in women’s literacy score accounted for by the EconomicClusters model was 5–12% less than the proportion of variance accounted for by the DHS Wealth Index model. The proportion of the variance in child HAZ and proportion of children who are deceased accounted for by the EconomicClusters model was similar to (0.4–2.5% less than) the proportion of variance accounted for by the DHS Wealth Index model. The EconomicClusters model requires asking only five questions, as opposed to greater than twenty Wealth Index questions. The EconomicClusters algorithm and app could facilitate health disparities research in any country with DHS data by generating ordinally-ranked, population-specific economic models that perform nearly as well as the Wealth Index in evaluating variability in health and social outcomes based on wealth status but that are more feasible to assess in time-constrained settings.

## Introduction

### The need for a new metric of economic status for health disparities research

Major public health and development organizations around the world, including the World Health Organization (WHO) [1], the United Nations Sustainable Development Goals program [2], and UNICEF [3], recognize health disparities as a major challenge to the realization of health goals for disadvantaged communities and for populations as a whole. Unsurprisingly but unacceptably, the poor around the world tend to have worse health outcomes than the wealthy [4]. In 2011, the WHO Commission on Social Determinants of Health developed five strategies aimed at decreasing global health disparities, the last of which was to “measure and understand the problem and assess the impact of action” [1]. Recognizing that the data necessary to monitor health disparities is severely limited in most of the world, the WHO recommends that this goal be accomplished by “establish[ing] national and global health equity surveillance systems” and prioritizing research measuring health disparities and the implementation of programs aimed at decreasing these disparities [1].

Health disparities research is complicated by the difficulty of measuring a person’s socioeconomic status (SES). Researchers use a variety of different variables as metrics of SES, but Braveman et al. (2005) argue that commonly used indicators of SES such as education and occupation should not be considered to be interchangeable with income or wealth-based metrics of economic status [5]. Braveman et al. (2005) give examples of the ways in which these variables exert different effects on health outcomes [5]. Regardless of a person’s economic wealth, his or her level of literacy and knowledge of health-related topics can influence his or her health [5]. Non-economic factors related to someone’s occupation, such as the amount of control they have over their work or the amount of manual labor they do, can also impact their health [5]. Furthermore, in their analysis of the 1999–2004 California Maternal and Infant Health Assessment dataset, Braveman et al. (2005) found significantly different odds ratios of receiving delayed or no prenatal care between different racial or ethnic groups depending on whether the results were adjusted for income, education, or both [5]. Because education and occupation affect health in different ways than do pure economic metrics, measuring education and occupation does not allow a researcher to directly answer questions about the association between wealth and health outcomes.

Determining an appropriate metric of economic status can also be challenging. Income can be difficult to assess in many low- and middle-income countries (LMICs) because trade in goods and services may be equally as important as monetary exchange, informal- or self-employment are common, and income may vary greatly depending on the availability of temporary work [4]. Other metrics of economic status, such as consumption or expenditures, require complex and time-consuming survey instruments or diaries to assess [4]. Assets-based models of economic status, such as the Demographic and Health Surveys (DHS) Wealth Index, are now commonly used for research in LMICs but also tend to require greater than twenty variables to calculate. It is possible to use such time-consuming metrics in cross-sectional survey studies where data collection must only be sustained for a short time. It is, in our experience, not feasible to spend the time that is necessary to collect data on so many economic questions in longitudinal emergency health surveillance databases, where data collection must be conducted quickly and sustained every day for years.

Some research groups have addressed this problem by using the same methodology the DHS uses to create the Wealth Index–a principal components analysis (PCA)–to generate a simplified Wealth Index consisting of fewer variables. Such simplified PCA-based methods must accept either relatively low agreement with the original Wealth Index or increasing numbers of variables to achieve higher levels of agreement with the original Wealth Index [6, 7, 8]. Furthermore, there are statistical limitations of the PCA model that make this an unfavorable option. Nonlinear relationships among variables can be misidentified in a PCA [9]. The Wealth Index model generally explains no more than 20 percent of the variance in the data when only the first Eigen vector is used [4]. Finally, the meaning of a PCA-based model may be difficult for policy-makers to understand in practical terms because it is merely the one linear combination of variables that explains the largest proportion of the variability among those variables, although these variables may actually be associated with one another in a non-linear fashion [10]. Wilunda et al. (2013), Pitchforth et al. (2007), and Patel et al. (2007) have proposed different simplified models of economic status that rely on subjective assessments of which variables are most important either by the researchers or by members of the target community and therefore are difficult to standardize for broad use in different settings [11, 12, 13]. None of these metrics has been widely adopted. If health equity surveillance systems are to be established in order to obtain the data necessary to tackle health disparities globally, then a simple, statistically sound, practically sensible, and internationally accepted metric of economic status must be established.

### Previous development of the EconomicClusters model

In order to address this need, we previously developed the EconomicClusters algorithm, which is a k-medoids clustering-based algorithm for generating simple models of economic status using limited numbers of DHS assets variables [14]. K-medoids is a widely used statistical algorithm that places individual observations in clusters based on how similar those individuals are to other individuals within a population. To use the EconomicClusters model, a researcher may first select the number of variables, *N*, that he or she would like to analyze in their survey instrument. Then, the EconomicClusters algorithm selects the *N* DHS Wealth Index variables that define the k-medoids clustering model consisting of the most distinct economic groups within a given DHS population. In this manner, a different model of economic status based on the most relevant assets for the specific DHS population being analyzed can be generated for each country with DHS data. By collecting data on these few assets variables for individuals in their surveys or disease registries, researchers can then compare the proportion of individuals from their new survey datasets from each economic group to the proportion of individuals in the nationally-representative DHS dataset from each economic group. Differences in the proportion of poor versus wealthy patients treated for a given disease in a survey dataset compared to the proportion of poor versus wealthy people in the overall population may indicate either different rates of that disease among different economic groups or differences in access to formal care for that disease process. Researchers can also compare health outcomes for individuals from different economic groups within their survey datasets to evaluate for health disparities. For a more detailed explanation of how the algorithm works, please see the Methods section.

We previously used the EconomicClusters model to define a five-asset metric of economic status for Cameroon [14]. These five questions have been asked in the new Cameroonian national trauma registry since 2015. The model will be used to assess for disparities in injury outcomes and access to trauma care. We have also developed an R statistical software package and R Shiny (web) application with a point-and-click user interface that is free and publicly-available on our website so that other researchers interested in measuring health disparities may also use our algorithm to define a metric of economic status for their country. They may then collect data on the variables that define their economic model either in cross-sectional surveys or in longitudinal disease-surveillance registries.

### Study aims

For a new economic metric to be widely adopted, it must first undergo tests of validity, meaning it must be shown to measure what it purports to measure. There are multiple different types of validity. Our first study addressed the statistical validity of our model by discussing the methodological development of the algorithm and confirming its success at identifying distinct economic groups in simulated datasets [14]. A more broad type of validation is known as construct validation [15]. To evaluate the validity of a theoretical construct, hypotheses may be made and tested regarding the association between the constructed metric and other measurable phenomena [15]. If the hypotheses hold true, then they provide evidence of the validity of the theorized metric [15]. In our case, the idea to be tested is that a population may be divided into distinct wealth groups based on access to a few assets, and that these wealth groups will be useful in assessing for health disparities based on economic status. If the algorithm does produce valid models of economic status for health disparities research, we hypothesize that the economic groups defined by the algorithm should correlate in a consistent manner with health and other socioeconomic outcomes that are widely accepted as being associated with wealth status. Strauss and Smith (2009) note that “validation is a process not an outcome” [15] meaning that a single study cannot validate a metric but can provide evidence of its validity [15]. The first aim of this study is to provide an initial assessment of the validity of the EconomicClusters model as a metric of economic status for health disparities research.

The second aim of this study is to optimize the utility of the EconomicClusters model and algorithm. Any new economic metric must be relatively simple to interpret such that policy-makers can act upon health disparities data obtained using the metric. We have received feedback that having as many as twenty economic groups can seem overwhelming when attempting to analyze the practical implications of disparities between different groups in the population. Our collaborators have suggested that the algorithm would be more practically useful if it generated economic models consisting of a smaller number of economic groups that were ranked objectively by the algorithm. Finally, the algorithm must be able to produce useful economic models in different contexts beyond Cameroon. To evaluate the utility of the model in another LMIC context, we develop and test the validity of an EconomicClusters model for Ghana.

By assessing the validity of the EconomicClusters model as a metric of economic status compared to socioeconomic and health outcomes with known economic disparities and by improving the user-friendliness and interpretability of the model, we aim to facilitate its use in time-constrained, low-resource settings around the world. As advocated by the WHO [1], if we are to eliminate health disparities, we must measure health disparities. Furthermore, if we want to increase the amount of research being done on health disparities, we must work to make the measurement of health disparities by economic status as sustainable as possible.

## Materials and methods

The data analyzed in this study were obtained from the DHS nationally representative cross-sectional survey datasets for Cameroon (2011) [16] and Ghana (2014) [17]. These datasets include all assets variables measured by the DHS to calculate the DHS Wealth Index, as well as other variables related to health and socioeconomic status. Missingness among all Wealth Index variables included in the EconomicClusters analysis for Cameroon and Ghana was less than two percent. Data analysis was conducted using RStudio version 1.0.143 [18] and the EconomicClusters RShiny app.

### Background methodology regarding the previous development of the EconomicClusters algorithm and its use in the Cameroonian national trauma registry

The previously developed EconomicClusters algorithm works as follows [14]. First, the researcher selects the number of assets questions they would like to ask in their survey or registry. For this example, we will develop an economic model based on five variables. The EconomicClusters algorithm defines every possible combination of five variables from all the DHS assets variables used to calculate the Wealth Index. For each combination of five assets, the algorithm runs the weighted k-medoids clustering algorithm from R package *WeightedCluster* [19] to place each household from the DHS dataset into a cluster based on their ownership of these five assets. K-medoids clustering requires that the researcher specify the number of clusters, but the optimal number of economic groups within a population is not known *a priori*. Thus, a range of cluster numbers must be evaluated in order to determine the optimal number of clusters. The EconomicClusters algorithm generates a k-medoids clustering solution for each number of clusters specified by the researcher for each combination of assets. The model consisting of the optimal set of assets and the optimal number of clusters is selected as the clustering model with the highest average silhouette width (ASW). ASW is calculated from a dissimilarity matrix. Each position on the matrix consists of the Gower’s distance measure of how dissimilar two observations from the overall database are to one another based on their answers to the five assets questions. ASW is a measure of how similar observations in a given cluster are to each other and how different they are from individuals in other clusters [20]. Thus, clustering solutions with a higher ASW consist of economic groups that are more distinct from each other based on their ownership of the five assets variables used to define that model. Researchers can ask the five questions that define the optimal model in their survey or disease registry.

When we ran the EconomicClusters algorithm on the 2011 Cameroonian DHS dataset, the clustering model with the highest ASW consisted of twenty economic clusters defined by the following five variables: rural versus urban setting, ownership of agricultural land, cell phone ownership, home financing, and cooking fuel type [14]. These five questions are asked in the Cameroonian national trauma registry. The function *EC_patient* from our R package *EconomicClusters* (available on Github) assigns registry participants to the EconomicCluster whose medoid gave the same answer as the registry participant did to the highest number of the five questions [21]. Medoids are the observations selected by the EconomicClusters k-medoids clustering-based algorithm to best define each of the EconomicClusters. If there is a tie in how similar the registry participants’ answers were to two different medoids, then the registry participant’s responses to the five model-defining questions are compared to the responses of all members of the DHS dataset. If the DHS households that responded most similarly to the registry participant all belong to the same EconomicCluster, then the registry participant is placed in that cluster. If not, as is particularly the case with missing registry data or very rare combinations of assets, then the registry participant cannot be assigned to an EconomicCluster. In this manner, a registry participant is assigned to the EconomicCluster with the DHS households that have the most similar EconomicClusters asset profile to the registry participant.

Once survey participants have been assigned to an EconomicCluster, the economic clustering groups can be used in two ways. In the newly collected dataset, differences in outcomes from injury or access to trauma care can be determined for the different economic groups defined by the EconomicClusters model. The proportion of individuals in the trauma registry that come from each economic group can also be compared to the proportion of individuals in the overall DHS dataset from each economic group. For example, if the poorest groups have limited access to hospital-based trauma care due to cost, there might be a smaller proportion of poor patients among those who present to the hospital for trauma care than in the overall DHS population. Before using the EconomicClusters model to evaluate for health disparities in the Cameroonian national trauma registry, we aim to optimize and evaluate the validity of the EconomicClusters model using the methods below.

### Descriptive statistics for Cameroonian economic groups

In order to provide the context necessary to interpret the wealth status of the Cameroonian economic groups defined previously [14], we calculated the cumulative proportion of households with each of the following assets: 1) urban versus rural location; 2) water source (piped into dwelling, obtained from a well, obtained from a natural source such as a stream, river, lake, etc.); 3) type of toilet facilities (flush toilet, pit latrine, no toilet); 4) cooking fuel (wood, charcoal, liquid petroleum gas (LPG)); 5) home financing (whether respondents own, rent, or live in their homes for free); and 6) ownership of the following assets: agricultural land, cell phone, electricity, radio, TV, motorcycle, car, bank account, stove, cd/dvd player, or computer. Only five of these variables—rural versus urban setting, ownership of agricultural land, cell phone ownership, home financing, and cooking fuel type—need to be asked to define the model. We also calculated the proportions of households in each economic group that possess the other asset variables listed above in order to provide further insight into the wealth of the different economic groups. The proposed group names were developed previously by our research group for the economic clusters [14] based on the assets available to the members of each group (e.g. clusters without cell phones were interpreted as being poorer than clusters with cell phones). Note that LPG is a more expensive fuel source than wood or charcoal.

### Analysis of child height-for-age Z-score, women’s literacy score, and proportion of children who are deceased by EconomicCluster for Cameroon

Three variables from the DHS dataset that are known to be associated with poverty are child height-for-age Z-score, women’s literacy rates, and child mortality. Child height-for-age Z-score is a marker of childhood malnutrition and has been shown to be associated with socioeconomic status in many different settings [22, 23, 24]. Although educational attainment measures a different aspect of socioeconomic status than wealth, educational markers such as literacy also tend to be correlated with pure economic metrics [5]. Disparities in rates of childhood mortality between poor and wealthier populations is a widely recognized global problem [25] and a target of action by international health organizations such as the WHO [26]. We assessed the association between the EconomicClusters groups and these three variables as follows. The weighted average child height-for-age Z-score for all children with available data in all households in each economic cluster was calculated. Women’s literacy levels are recorded in the DHS individual (women’s) dataset on a scale from 0 to 2 with 0 being “Cannot read at all”, 1 being “Able to read only parts of a sentence”, and 2 being “Able to read whole sentence” [16]. Weighted average literacy scores based on this scale for women from each cluster were calculated. Finally, as a measure of child mortality, the proportion of a woman’s children who are deceased was calculated for the women from the DHS individual (women’s) dataset. For each woman survey respondent, the number of deceased sons plus the number of deceased daughters divided by her total children ever born were calculated. Weighted mean proportion of children who are deceased was calculated for each economic cluster.

### Combination of twenty Cameroonian EconomicClusters into fewer economic groups using agglomerative hierarchical clustering

Agglomerative hierarchical clustering was used to combine the twenty Cameroonian EconomicClusters into fewer economic groups in order to make the model more easily interpretable. The theory behind the approach is that economic groups that experience different levels of health or socioeconomic outcomes must be kept separate in order to appropriately measure health disparities between groups. However, economic groups that have different types of wealth in terms of which assets are available to the members of these groups but which have similar health and socioeconomic outcomes may be combined together into a broader wealth bracket for the sake of measuring the success of programs aimed at improving health outcomes among vulnerable groups.

Agglomerative hierarchical clustering is a widely-used unsupervised clustering-based methodology that sequentially collapses observations within a dataset into fewer and fewer clusters based on how similar or dissimilar observations are to other observations within the dataset [27]. Advantages to this method include that the algorithm does not need to be provided *a priori* with a known number of population clusters and that the resulting clustering tree demonstrates the degree of similarity of clusters to one another [27]. Agglomerative hierarchical clustering was conducted separately for rural and urban populations. This practice is consistent with the DHS approach of defining separate Wealth Index scales for rural and urban populations given that wealth may manifest as different assets among rural and urban groups [28].

We then had to define some function of these variables to compare among clusters. We chose to compare the average of each variable, though we note that other metrics could similarly have been used. The values of mean child height-for-age Z-score, women’s literacy, and proportion of children who are deceased for each of the twenty Cameroonian EconomicClusters were calculated and scaled in order to prevent variables with larger values from dominating the model. Then, a dissimilarity matrix was calculated for the differences in cluster means of these variables using function *daisy* from R package *cluster* [29] with the default Euclidean distance metric. Starting with cluster means allowed the hierarchical clustering algorithm to begin clustering with the known twenty economic groups rather than individual observations. Agglomerative hierarchical clustering was run on this dissimilarity matrix using function *hclust* from R package *stats* [30] for rural and urban populations. The resulting cluster dendrograms were then used to define a model of fewer economic groups each consisting of multiple of the twenty original EconomicClusters.

### Definition of an EconomicClusters model for Ghana from the DHS 2014 dataset

An EconomicClusters model was developed for Ghana using the DHS 2014 dataset [17] and the EconomicClusters package available for R on Github [21]. The methodology behind this k-medoids clustering-based algorithm was previously described in detail in Eyler, et al. [14] and is reviewed in Section 2.0 above. We opted to create a model consisting of rural versus urban setting and four of the other DHS assets variables that were available to at least 10% of the Ghanaian population. We chose to eliminate assets available to less than 10% of the population because the goal of the optimized algorithm is to define few broad economic groups whereas groups defined by rare assets would consist of a relatively small proportion of the overall population. In order to develop an economic model with fewer clusters for the ease of interpretation, we re-coded the algorithm with the option to select the smallest number of clusters, *K*, from five to twenty that included a model with an average silhouette width of at least 0.70. This threshold has been proposed by Kauffman and Rousseau for defining strong clusters within a dataset [31]. As noted previously, a higher ASW means that the economic groups are more distinct from one another [20]. Our updated EconomicClusters algorithm [21] can select the economic model consisting of the asset combination that defines *K* population clusters with the highest average silhouette width. This threshold ASW option is now a standard part of the EconomicClusters package [21].

For each of the Ghanaian economic groups defined by the algorithm, weighted proportion of each economic group with the five model-defining assets and other relevant assets from the DHS dataset were calculated, including: rural versus urban setting, cell phone ownership, computer ownership, type of cooking fuel, type of flooring material, type of toilet facility, access to the internet, and ownership of bicycles, radio, color TVs, refrigerators, and DVD players.

### Ordinal ranking of Cameroonian and Ghanaian economic groups based on child height-for-age Z-score, women’s literacy score, and proportion of children who are deceased and comparison to DHS wealth index quintiles

Because the EconomicClusters model is based on unsupervised clustering, the model does not intrinsically rank groups based on their wealth status. Subjectively, it is intuitive that an economic group whose defining characteristics were an urban population without cell phones who cook with wood fuel would have a lower economic status than an urban population with cell phones who cook with liquid petroleum gas (a more expensive type of fuel). However, comparing the economic groups by outcome variables known to be associated with wealth status (such as child height-for-age z-score, women’s literacy, and child mortality) allows for an objective method of ordinally ranking the economic groups that does not rely on an individual researcher’s interpretation of group wealth. Weighted mean values of child height-for-age Z-score, women’s literacy score, and proportion of children who are deceased were calculated for each of the Cameroonian and Ghanaian EconomicClusters. Weighted mean values for child height-for-age Z-score, women’s literacy score, and proportion of children who are deceased were also calculated for the five DHS Wealth Index quintiles for Cameroon and Ghana. The DHS survey weights, defined as the inverse probability of household selection, were used for these calculations. Standard errors of these means were calculated and represented by error bars. We are concentrating on clustering based on the marginal means of covariates within clusters, but one could also use other appropriate measures (with appropriate accounting for differences in scale) like standard deviations and other moments. A one-way analysis of variance (ANOVA, using R package *car* [32]) was conducted to determine whether there were significant differences in group means for these three variables between the EconomicClusters groups and between the DHS Wealth Index Quintiles for Cameroon and Ghana. Values of eta squared were calculated (using R package *DescTools* [33]) to determine the proportion of variance in child HAZ, women’s literacy score, and proportion of children who are deceased accounted for by EconomicCluster and by DHS Wealth Index quintile. Two-sample student’s t-tests were run to determine the statistical significance of the difference between mean values of these variables for the adjacently-ranked rural and urban groups for the EconomicClusters and between adjacently-ranked DHS Wealth Index quintiles. Rural and urban groups were analyzed separately for the EconomicClusters groups in keeping with DHS practices for analyzing wealth-based economic status where separate rural and urban wealth indices are encouraged [28]. The R package *survey* [34] was used for the calculation of weighted means, proportions, standard errors, ANOVA, eta squared, and t tests to take into account the complex survey design of the DHS datasets. The ordinal ranking of the Cameroonian and Ghanaian EconomicClusters was assigned based on the relative cluster mean levels of child HAZ, women’s literacy score, and proportion of children who are deceased.

## Results

**Fig 1:**
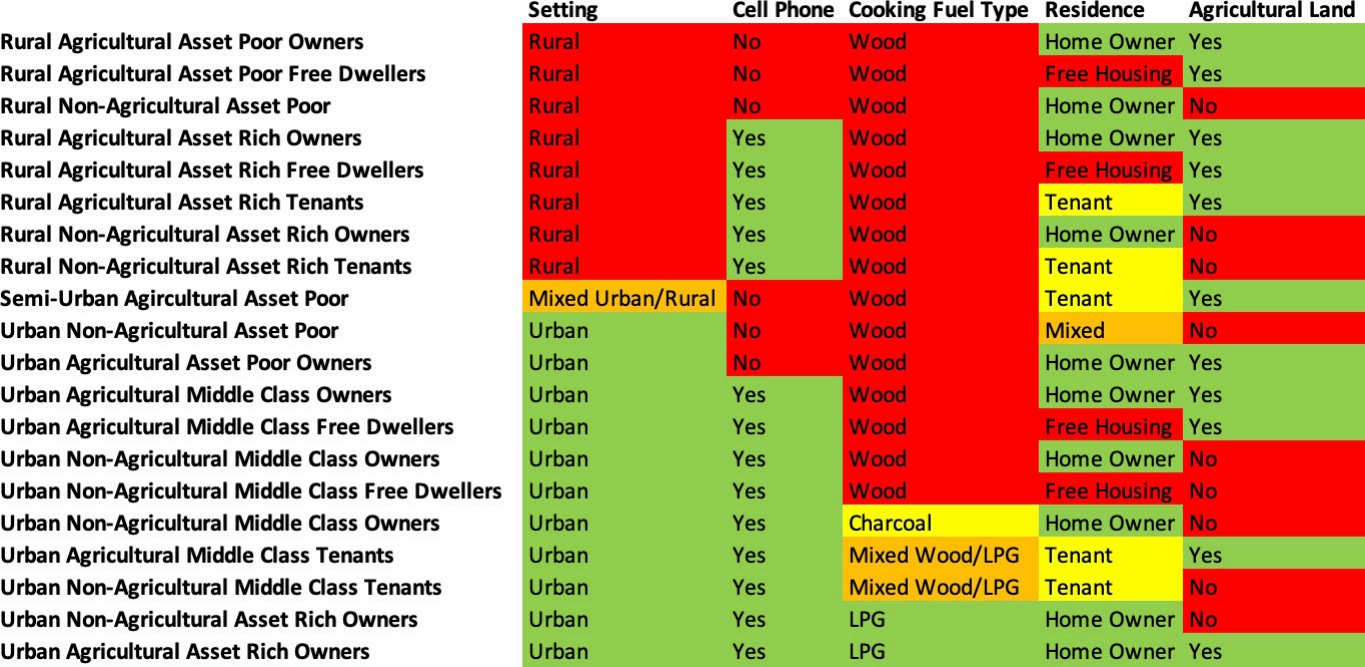
Most common defining assets of twenty Cameroonian EconomicClusters and proposed cluster interpretations. Caption: For more details regarding the development of this model, please see Eyler et al. [14].

**Fig 2:**
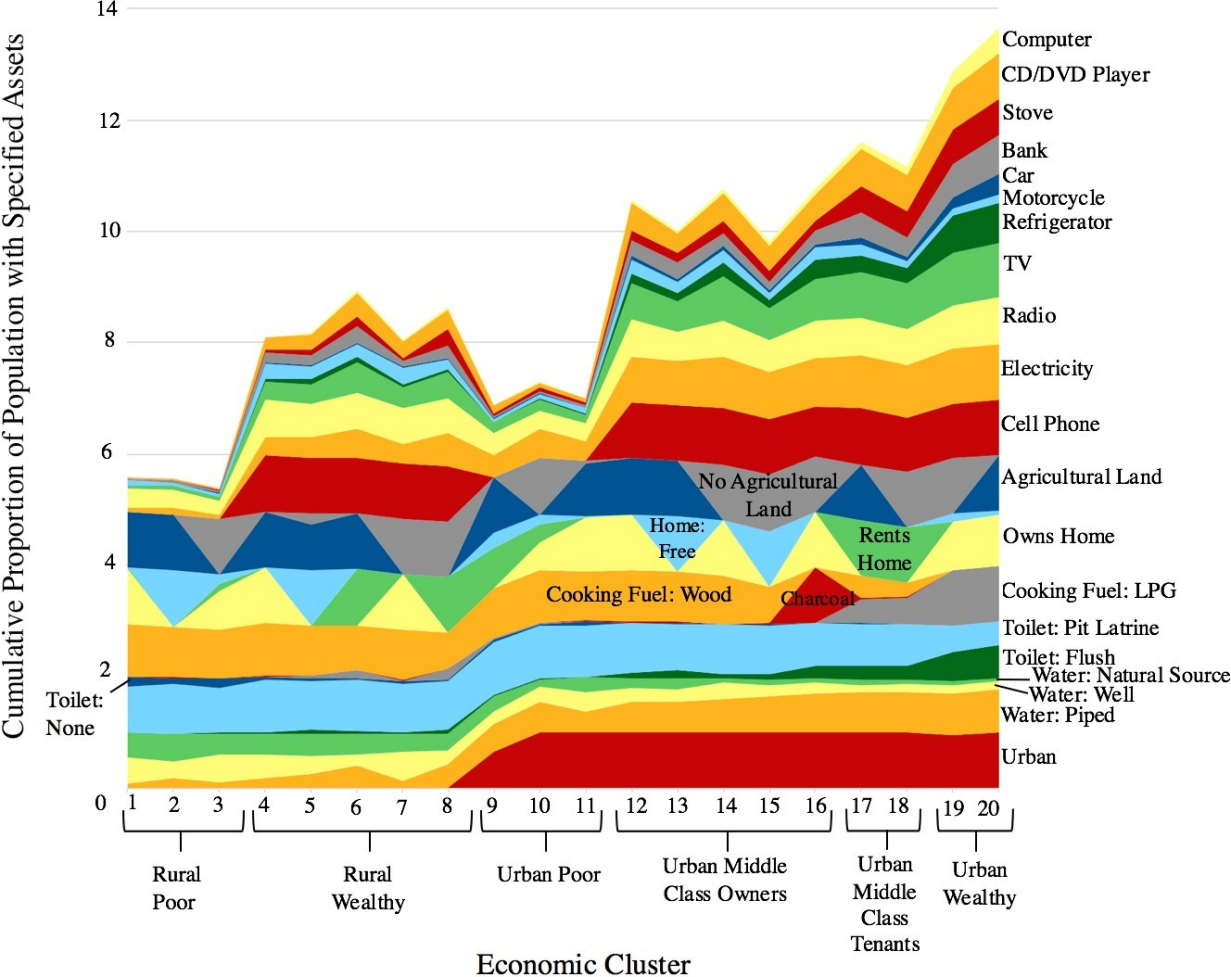
Distribution of various DHS Wealth Index assets among twenty Cameroonian EconomicClusters.

The EconomicClusters model for Cameroon developed by Eyler et al. [14] consists of twenty economic clusters defined by the following five assets variables: rural versus urban setting, agricultural land ownership, cell phone ownership, whether a household owns, rents, or lives in their home for free, and type of fuel used for cooking. Fig 1 shows the most common answers to each of the five model-defining assets questions given by households within each cluster, as previously described in Eyler et al. [14]. When naming the clusters, clusters with cell phones were considered wealthier than clusters without cell phones, and clusters who cook with LPG (a more expensive fuel source) were considered wealthier than clusters who cook with wood or charcoal. Fig 2 shows the cumulative proportion of each cluster population with each of the five model-defining assets and other DHS Wealth Index assets. The relative wealth of the different clusters was evaluated based on the cumulative proportion of assets (both the five model-defining assets and other DHS Wealth Index assets) available to households in those clusters. For the sake of this simplified interpretation, clusters comprised of individuals who either own their homes or live in them for free were described as “owners”. The Cameroonian DHS 2014 dataset consisted of 14,214 households. 13,768 of these households had no missing data for any of the Wealth Index variables evaluated as possible components of the EconomicClusters model.

**Fig 3:**
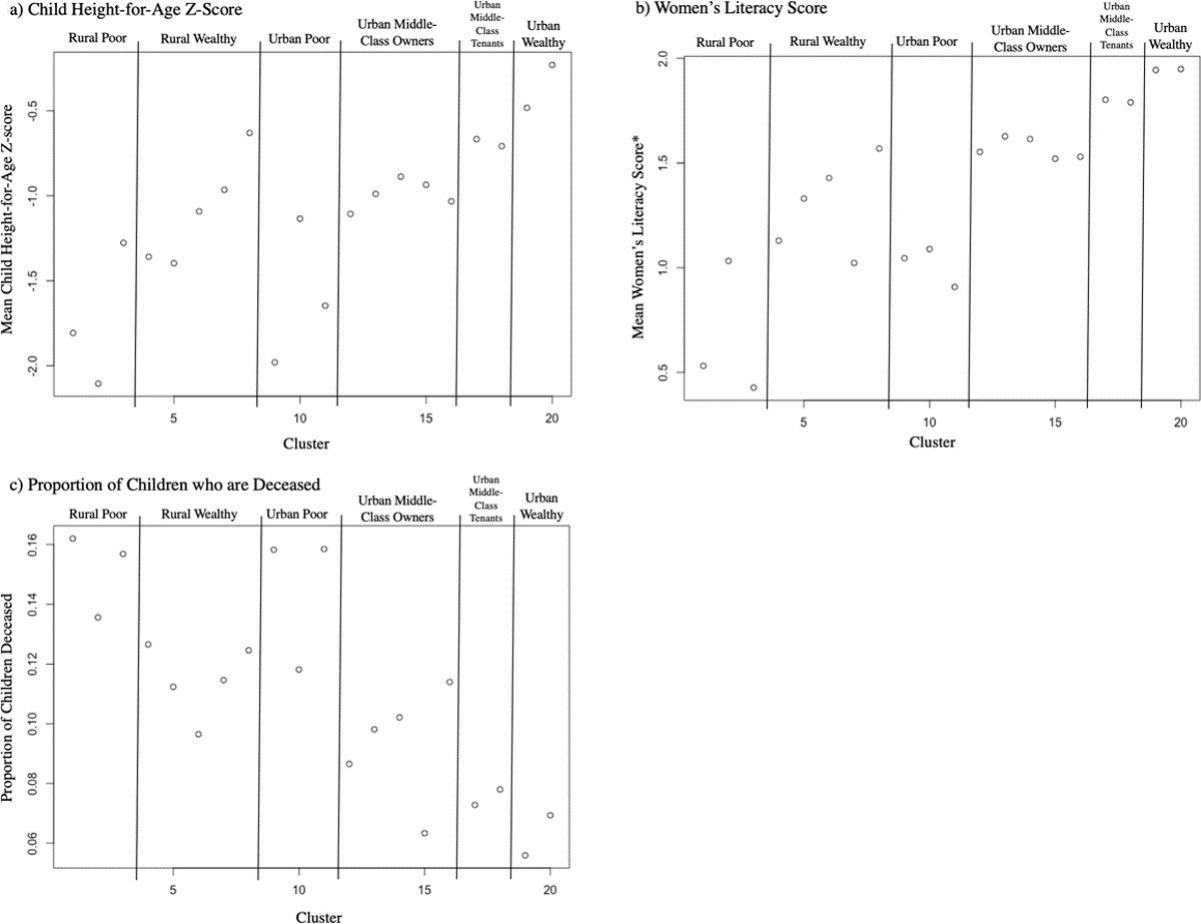
Mean child height-for-age Z-score, women’s literacy score, and proportion of children who are deceased by Cameroonian EconomicCluster. Caption: †Literacy Score: 0 = “Cannot read at all”, 1 = “Able to read only parts of a sentence”, 2 = “Able to read whole sentence”.

Data regarding child height-for-age Z-score was available for 5912 children living in the surveyed households. Data regarding women’s literacy level and proportion of children who are deceased was available for 15169 and 11023 of the 15426 women from the Cameroonian women’s dataset, respectively. As shown in Fig 3, mean child height-for-age Z-score, women’s literacy level, and proportion of children who are deceased tend to be associated in the manner expected with the initially proposed interpretations of the relative wealth of the twenty EconomicClusters, although there is some variability across the three variables in certain groups. Note also that multiple clusters have very similar levels of these socioeconomic and health outcomes.

**Fig 4:**
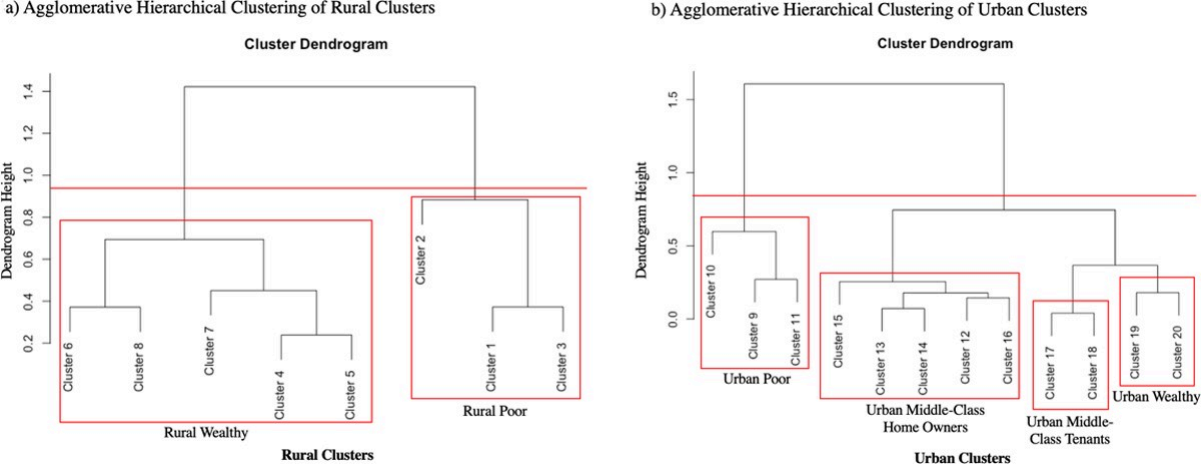
Agglomerative hierarchical clustering of Cameroonian rural and urban EconomicClusters based on mean scaled child height-for-age Z-score, women’s literacy score, and proportion of children who are deceased.

Fig 4 shows the results of agglomerative hierarchical clustering of the rural and urban Cameroonian EcnomicClusters based on mean scaled child height-for-age Z-score, women’s literacy scores, and proportion of children who are deceased. For ease of interpretation, we selected a cutoff level of height on the cluster dendrograms that would produce a total of five to ten broad economic groups (demarcated by red lines). Given preliminary data analysis of a large household survey from Cameroon suggesting that LPG use is an important economic variable in predicting access to health services, we elected to keep clusters 19 and 20 (who use LPG) separate from clusters 17 and 18 (who use some wood and some LPG). Of note, the cluster combinations defined objectively by the agglomerative hierarchical clustering algorithm agreed with our initial subjective impressions of the relative wealth status of the twenty original clusters.

Table 1 demonstrates the results of one-way ANOVA evaluating mean differences in child height-for-age Z-score, women’s literacy score, and proportion of children who are deceased across both the simplified Cameroonian EconomicClusters and the Cameroonian DHS Wealth Index quintiles. P-values for the ANOVA F statistic were less than 2.2 x 10^−16^ for all dependent and independent variables for Cameroon. Eta squared values demonstrate that the DHS Wealth Index accounted for approximately 9% of the variance in child height-for-age Z-score, 35% of the variance in women’s literacy score, and 5% of the variance in proportion of children who are deceased. EconomicCluster accounted for approximately 6% of the variance in child height-for-age Z-score, 23% of the variance in women’s literacy score, and 4% of the variance in proportion of children who are deceased.

**Table 1:** Results of ANOVA and eta squared comparing proportion of variance in child height-for-age Z-score, women’s literacy score, and proportion of children who are deceased accounted for by Cameroonian DHS Wealth Index and EconomicClusters models.

Variable	Economic Metric	ANOVA p-value	Eta Squared
Child Height-for-Age Z-Score			
	Wealth Index	<2.2 x 10^−16^	0.086
	Economic Cluster	<2.2 x 10^−16^	0.061
Women's Literacy Score			
	Wealth Index	<2.2 x 10^−16^	0.349
	Economic Cluster	<2.2 x 10^−16^	0.225
Proportion of Children Deceased			
	Wealth Index	<2.2 x 10^−16^	0.053
	Economic Cluster	<2.2 x 10^−16^	0.043

**Fig 5:**
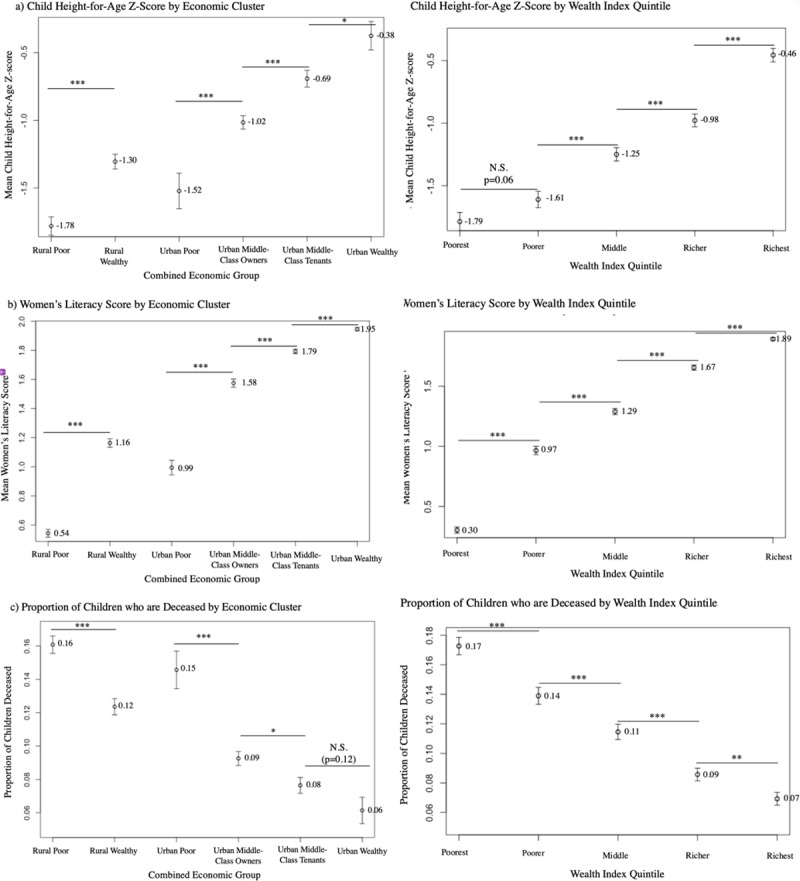
Mean child HAZ, women’s literacy score, and proportion of children who are deceased for combined Cameroonian EconomicClusters and DHS Wealth Index quintiles. Caption: Horizontal bar indicates p-value for two-sample student’s t-tests between adjacent groups: * = p<0.05, ** = p<0.01, *** = p<0.005 †Literacy Score: 0 = “Cannot read at all”, 1 = “Able to read only parts of a sentence”, 2 = “Able to read whole sentence” Error Bars: Standard error of the mean.

Fig 5 shows the average child height-for-age Z-score, women’s literacy scores, and proportion of children who are deceased for each of the six combined economic groups defined by the agglomerative hierarchical clustering model, as well as for the five DHS Wealth Index quintiles. Final ordinal wealth rankings for the rural and urban Cameroonian EconomicClusters were determined based on increasing levels of mean child HAZ and women’s literacy score and decreasing rates of child mortality. The differences between successively ranked EconomicClusters within the rural and urban populations were statistically significant (p<0.05) across all three variables, except for the proportion of deceased children between the “Urban Middle-Class Tenants” and the “Urban Wealthy”. The differences between successively ranked DHS Wealth Index quintiles were statistically significant (p<0.05) across all three variables, except for mean child HAZ between the “Poorest” and the “Poorer” quintiles.

**Fig 6:**
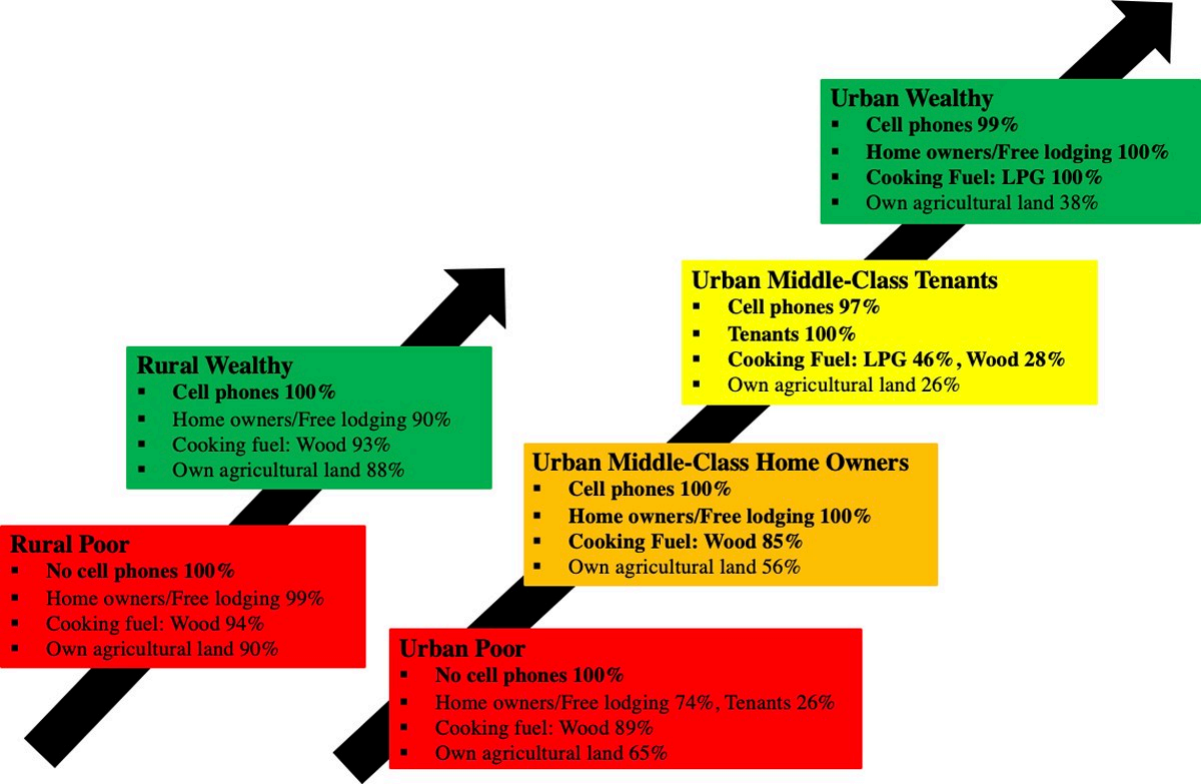
Simplified ordinal economic model for Cameroon.

Fig 6 demonstrates the simplified, ordinally-ranked economic model for Cameroon based on two rural and four urban economic groups. Such a graphic could be presented to policy-makers in order to explain the meaning of the economic metric when discussing the results of health disparities research.

The Ghanaian DHS 2014 dataset consisted of 11,835 households. 11,827 of these households had no missing data for any of the Wealth Index variables evaluated as possible components of the EconomicClusters model. When the EconomicClusters algorithm was run for the Ghanaian population from the DHS 2014 Ghana dataset with a threshold criterion of selecting the minimum number of clusters from 5 to 20 with an average silhouette width of at least 0.70, the algorithm produced an economic model with five clusters–two rural, and three urban. The asset variables selected were rural versus urban setting (chosen to be included in all possible models), cell phone ownership, computer ownership, cooking fuel, and floor material. The most common floor material was cement for all five clusters, but the second most common floor material varied among the groups The average silhouette width of this model was 0.88, which is well above our threshold level. Cluster 1 is defined by the majority of the population being rural, having no cell phones or computers, cooking with wood (82%), and having 73% cement floors and 17% earth floors. Cluster 2 is defined by the majority of population being rural, having cell phones but no computers, cooking with wood (63%) or charcoal (24%), and having 70% cement floors and 13% linoleum or rubber floors. Cluster 3 is defined by the majority of the population being urban, having no cell phones or computers, cooking with charcoal (49%) or wood (40%), and having 82% cement floors and 5% earth floors. Cluster 4 is defined by the majority of the population being urban, having cell phones but no computers, cooking with charcoal (48%) or LPG (32%), and having 61% cement floors and 15% carpet floors. Cluster 5 is defined by the majority of the population being urban, having computers and cell phones, cooking with LPG fuel (70%), and having 37% cement floors and 31% tile floors.

**Fig 7:**
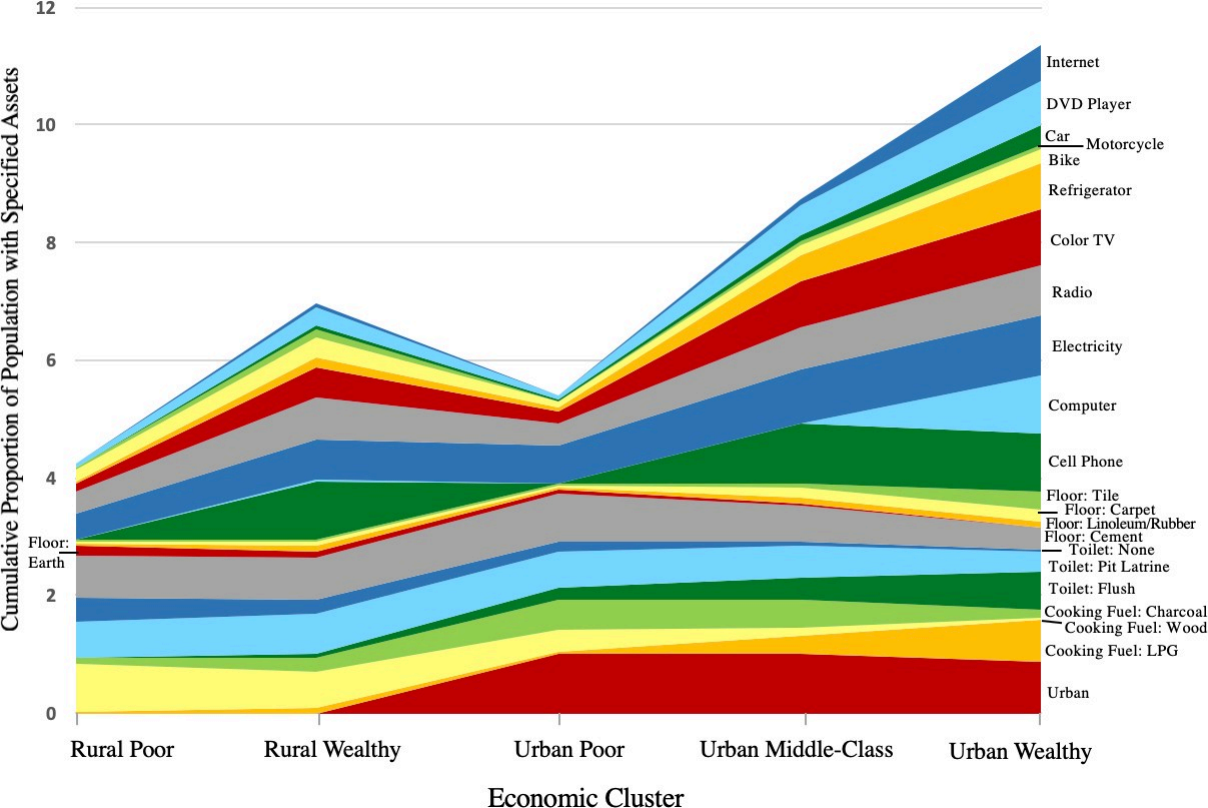
Cumulative proportion of various DHS Wealth Index assets among Ghanaian EconomicClusters.

In order to assist with the interpretation of the relative wealth of the different clusters, availability of other DHS assets variables are also reported. Fig 7 shows the cumulative proportion of different DHS assets including each of the model-defining asset variables for the five Ghanaian EconomicClusters. The distribution of assets variables within each EconomicCluster suggests the relative wealth status of these clusters, though they have not yet been objectively ranked by the algorithm.

As with the Cameroonian EconomicClusters model, the cluster averages of child height-for-age Z-score, women’s literacy scores, and proportion of children who are deceased suggest an objective ordinal ranking system for the Ghanaian EconomicClusters. Information on child height-for-age Z-score was available for 3078 children from the surveyed households. Information on women’s literacy score and the proportion of a woman’s children who are deceased was available for 9377 and 6511 of the 9396 women from the Ghanaian individual dataset.

Table 2 demonstrates the results of one-way ANOVA evaluating mean differences in child height-for-age Z-score, women’s literacy score, and proportion of children who are deceased across both the Ghanaian EconomicClusters and the Ghanaian DHS Wealth Index quintiles. P-values for the ANOVA F statistic were less than 6 x 10^−5^ for all dependent and independent variables for Ghana. Eta squared values demonstrate that the DHS Wealth Index accounted for approximately 14% of the variance in child height-for-age Z-score, 17% of the variance in women’s literacy score, and 8% of the variance in proportion of children who are deceased. EconomicCluster accounted for approximately 12% of the variance in child height-for-age Z-score, 12% of the variance in women’s literacy score, and 8% of the variance in proportion of children who are deceased.

**Table 2:** Results of ANOVA and eta squared comparing proportion of variance in child height-for-age Z-score, women’s literacy score, and proportion of children who are deceased accounted for by Ghanaian DHS Wealth Index and EconomicClusters models.

Variable	Economic Metric	ANOVA p-value	Eta Squared
Child Height-for-Age Z-Score			
	Wealth Index	<2.2 x 10^−16^	0.135
	Economic Cluster	1.03 x 10^−14^	0.120
Women's Literacy Score			
	Wealth Index	<2.2 x 10^−16^	0.167
	Economic Cluster	<2.2 x 10^−16^	0.117
Proportion of Children Deceased			
	Wealth Index	1.22 x 10^−8^	0.082
	Economic Cluster	5.23 x 10^−5^	0.078

**Fig 8:**
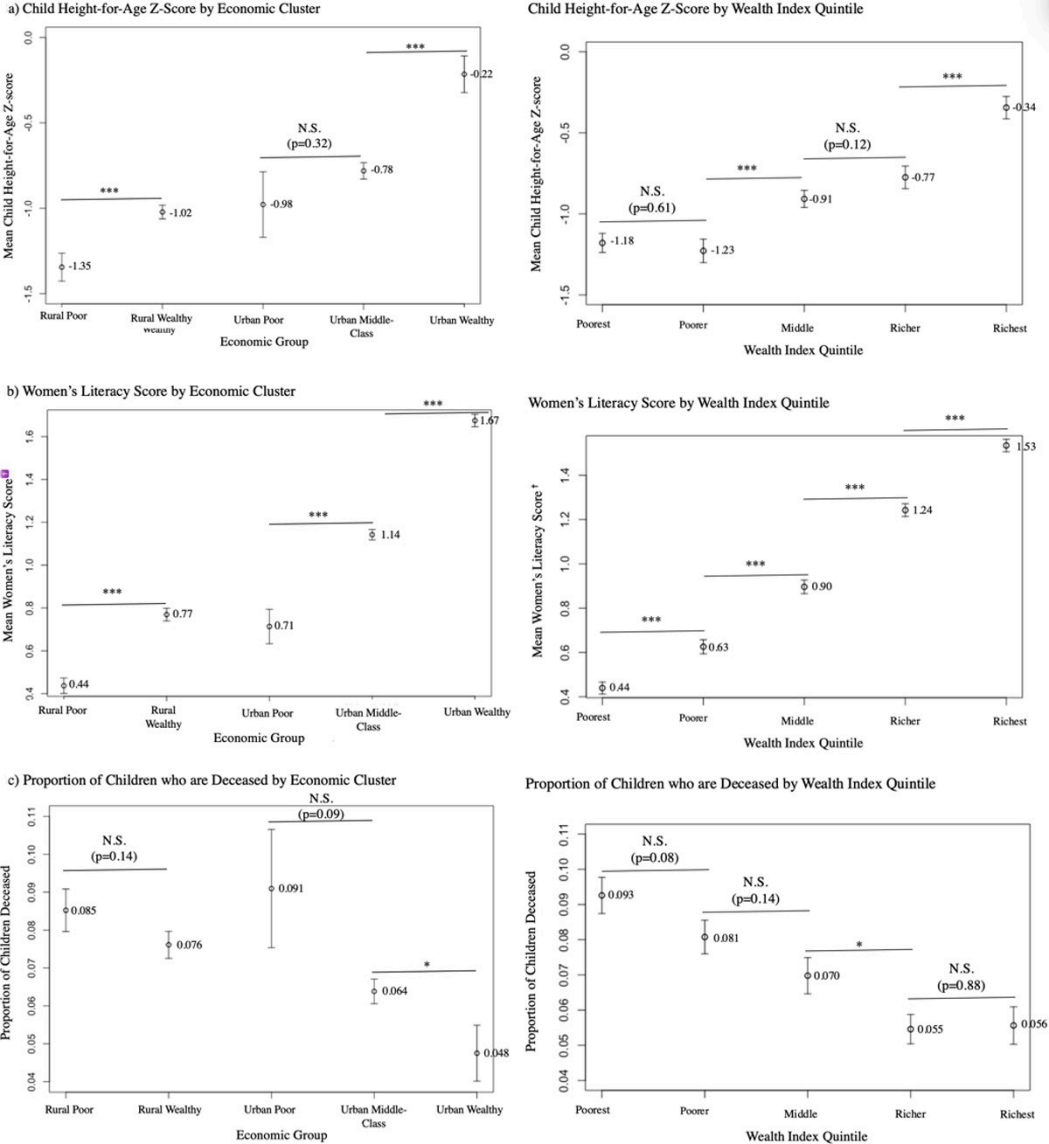
Mean Child HAZ, women’s literacy score, and proportion of children who are deceased for Ghanaian EconomicClusters and DHS Wealth Index quintiles. Caption: Horizontal bar indicates p-value for two-sample student’s t-tests between adjacent groups: * = p<0.05, ** = p<0.01, *** = p<0.005 †Literacy Score: 0 = “Cannot read at all”, 1 = “Able to read only parts of a sentence”, 2 = “Able to read whole sentence” Error Bars: Standard error of the mean.

Fig 8 shows the average household child height-for-age Z-score, women’s literacy scores, and proportion of children who are deceased for each of the Ghanaian EconomicClusters and for the Ghanaian DHS Wealth Index quintiles. The relative means of each of these variables for the Ghanaian EconomicClusters is consistent with our initial subjective interpretation of the wealth ranking of the rural and urban EconomicClusters. The differences in mean child height-for-age Z-score are statistically significant (p<0.05) between the “Rural Poor and Rural Wealthy” and between the “Urban Middle-Class” and “Urban Wealthy”, though not between the “Urban Poor” and “Urban Middle-Class”. The differences in mean literacy score were statistically significant across all adjacent rural and urban economic groups. The difference in the proportion of children who are deceased, however, was only statistically significant between the “Urban Middle-Class” and “Urban Wealthy”, although the mean values of this variable for each of the EconomicClusters was still consistent with the expected ordinal ranking for the rural and urban groups. For the Ghanaian Wealth Index quintiles, the differences in mean child height-for-age Z-score are statistically significant (p<0.05) between the “Poorer” and “Middle” quintiles and “Richer” and “Richest” quintiles though not between the “Poorest” and “Poorer” and “Middle” and “Richer” quintiles. The differences in mean literacy score were statistically significant across all adjacent quintiles. The difference in the proportion of children who are deceased was only significant between the “Middle” and “Richer” quintiles.

**Fig 9:**
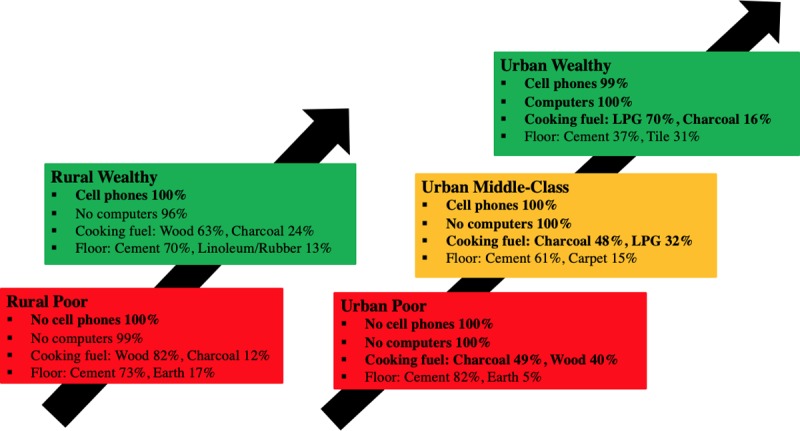
Simplified ordinal EconomicClusters model for Ghana.

Fig 9 demonstrates a simplified, ordinally-ranked EconomicClusters model for Ghana based on two rural and three urban economic groups. Such a model could be presented to policy-makers in order to explain the meaning of the economic metric when discussing the results of health disparities research.

## Discussion

In order for the EconomicClusters model to be a valid, useful, widely accepted metric of economic status, it must meet at least three primary criteria beyond its previously demonstrated statistical validity. It must measure an aspect of economic status that is consistently relevant for measuring health disparities. It must be practically simple to use and interpret such that policy-makers may act upon results obtained using this metric. Finally, it must be applicable to different LMIC contexts. Our results demonstrate the following, with these objectives in mind. Agglomerative hierarchical clustering of the twenty Cameroonian EconomicClusters based on cluster mean child height-for-age z-score (a marker of malnutrition), women’s literacy score, and the proportion of children who are deceased generated two rural and four urban economic groups. The simplified economic groups correlated consistently with increasing child HAZ and women’s literacy and with decreasing child mortality. The EconomicClusters model developed for Ghana using a threshold minimum ASW to define an economic model with fewer clusters was also associated with child height-for-age z-score, women’s literacy score, and the proportion of children who are deceased in a consistent manner. The differences in group mean outcome of child HAZ, women’s literacy score, and proportion of children who are deceased were statistically significant for similar numbers of groups between the EconomicClusters models and DHS Wealth Index models for Cameroon and Ghana. The proportion of variance in women’s literacy score accounted for by the EconomicClusters models was 5–12% less than the proportion of variance accounted for by the DHS Wealth Index models. The proportion of the variance in child HAZ and proportion of children who are deceased accounted for by the EconomicClusters models was similar to (0.4–2.5% less than) the proportion of variance accounted for by the DHS Wealth Index models. The fact that the Ghanaian EconomicClusters model performs almost as well as the DHS Wealth Index quintiles in its association with child HAZ, women’s literacy scores, and proportion of children who are deceased suggests that the model’s success in Cameroon was not merely a population-specific chance occurrence but one that we have replicated in another setting. We discuss both the implications and limitations of each of these results.

A methodology for condensing the twenty Cameroonian EconomicClusters into a smaller number of economic groups must determine which of the groups are practically more or less similar in terms of their socioeconomic status or vulnerability to health disparities. Groups that have similar levels of socioeconomic and health outcomes can then be analyzed together to assess for improvements in these outcomes with interventions aimed at decreasing these disparities. Agglomerative hierarchical clustering provides a statistically agnostic method for combining economic groups that also makes practical sense (for example, the hierarchical clustering model combined all groups without cell phones together in both the rural and urban populations) but does not rely on human interpretation. One limitation of this model is that it can only incorporate health, social, and economic variables that are available in the DHS dataset. The reason to use DHS variables to define the EconomicClusters model is that the DHS dataset contains nationally-representative data regarding the proportions of households in the population who have the specified assets. When the model is used to collect new data, for example as in a trauma registry, the economic status of the research subjects can be compared to the economic status of the overall population from the nationally-representative DHS datasets. Another limitation is that it produces a model with less complexity in rural populations than in urban populations. The DHS addressed this problem with their original Wealth Index by adding variables that were more likely to correspond to rural wealth and by calculating separate wealth indices for rural and urban populations [28]. However, this adds more variables to the overall model, and many groups who use PCA-derived Wealth Index models using DHS data simply generate one model for the entire population without distinguishing between rural and urban groups [35, 36, 37]. Despite these limitations, the finalized Cameroonian economic model revealed significant disparities in our three health and social outcome variables, suggesting that the EconomicClusters model would likely be useful for measuring health disparities by economic status across a broader range of health outcomes, as well. Next steps for using the model will include measuring health disparities in the new Cameroonian national trauma registry. Data on the five DHS assets variables included in the Cameroonian model have been collected in this registry since 2015, demonstrating the feasibility of longitudinal data collection using the EconomicClusters model.

The Ghanaian EconomicClusters model demonstrates the applicability of the algorithm to another LMIC context. The mean levels of child height-for-age z-score, women’s literacy score, and the proportion of children who are deceased are also associated with Ghanaian economic group in a way that makes practical sense based off of the assets available to each group. For example, these outcomes are, on average, worse among groups without cell phones and without computers than among groups with these assets. The mean differences in outcomes were statistically significant for all adjacently-ranked Ghanaian EconomicClusters and Wealth Index quintiles for women’s literacy scores but only for some of the adjacently-ranked groups for child HAZ and proportion of children who are deceased. This lack of statistical significance between some of the EconomicClusters and DHS Wealth Index quintiles could be due to a smaller overall disparity in these outcomes in Ghana compared to Cameroon. For example, rates of under-five mortality in Ghana are lower than in Cameroon at 59 versus 80 deaths per 1,000 live births [38]. Because the overall range of child mortality between the poorest and wealthiest groups was smaller in Ghana than in Cameroon, the differences between adjacent groups may simply not have been enough to achieve statistical significance with the amount of within group variability demonstrated for this outcome. Despite this lack of statistical significance between some of the groups, the ordinal ranking of economic groups suggested by all three variables was the same.

One would not necessarily expect that a valid economic model would show statistically significant differences between all groups for all potential outcome variables. To better determine the relevance of the statistical significance or lack thereof between adjacent EconomicClusters, we compared the eta squared values demonstrating the proportion of variance in these three variables accounted for by the EconomicClusters and DHS Wealth Index models for Cameroon and Ghana. The eta squared values suggest that the Wealth Index model accounts for slightly more variance (0.4–2.5%) in child HAZ and proportion of children who are deceased and more variance (5–12%) in women’s literacy score compared to the EconomicClusters model. Given that the EconomicClusters model requires asking many fewer questions to evaluate compared to the DHS Wealth Index model, we find this to be an acceptable difference in the proportion of variance accounted for by the two models.

One methodological consideration for use of the model is that there are advantages and disadvantages to starting with a model consisting of more clusters and condensing them compared to beginning with a model consisting of fewer clusters. In the Cameroonian model, certain model variables turned out to be important in the condensed model at only certain levels of economic status but not others. For example, home ownership versus tenancy was only important in distinguishing the urban lower- and upper-middle class groups, but these two groups have statistically significant differences in our three socioeconomic and health outcomes. A model that begins with fewer groups does not allow for as nuanced relationships between the different assets variables across groups. Despite that potential advantage to beginning with a model with more clusters, the goal of the EconomicClusters model is not to find the one correct economic grouping within a population but rather to define one statistically and practically valid economic grouping that can be used consistently over time to track health disparities. One option would be to create two models for a given country and let policy-makers or researchers choose which model to use long-term. As the Ghanaian model defines five economic categories that correlate with our three socioeconomic and health indicators in a practical manner, this model has been selected for Ghana and will be further assessed in a Ghanaian survey of the impact of health insurance on health outcomes.

In this manner, we follow the same approach to the development, validation, and evolution of the EconomicClusters model as the DHS took for the development, validation, and evolution of the Wealth Index. First, the PCA-based Wealth Index methodology was proposed by Filmer and Pritchett [39] and then modified by Rutstein and colleagues over time in order to address limitations of the model [28, 40, 41]. The DHS assessed the performance of the Wealth Index in distinguishing different economic groups based on variables known or suspected to be associated with economic status, including fertility rates, use of medical prenatal care, child mortality rates, rates of childhood stunting, and education levels [40]. Then, the DHS and subsequently many other research groups began to use the Wealth Index model in studies aimed at measuring health disparities which over time continued the validation process [35, 36, 37, 42]. Our work in Cameroon and our collaborators’ work in Ghana will continue this process for the EconomicClusters model. Other research groups interested in using the model or developing a new model for their country may download the free EconomicClusters app from our website and may continue the process of ongoing model assessment and validation in different contexts.

The aim of this study is to define a useful and valid metric of economic status, not to define one best or all-encompassing metric of economic status. As Paula Braveman points out in her article “Socioeconomic Status in Health Research: One Size does not Fit All”, there are many socioeconomic metrics that measure related but different aspects of socioeconomic status [5]. It is important to understand what any given metric is assessing and when it is appropriate to use that metric [5]. Assets-based indices in general measure aspects of a household’s material wealth and standard of living [4]. The advantages of assets-based metrics of economic status include that, compared to consumption and expenditures, these metrics may be more stable over time, easier to assess, and are available in many datasets [4]. Nationally-representative DHS data is available in over 90 countries. Population-specific EconomicClusters models consisting of different DHS assets variables could thus be generated for any of these countries using existing, publicly-available data to ensure that the economic models are relevant to the cultures in which they are being used. The DHS datasets are periodically updated with new surveys, which allows for the comparison of study data to DHS population-level data over time.

The disadvantages of assets-based metrics include that the availability of certain assets may change in a population over time [4]. However, so long as the DHS continues to measure the same assets periodically, then the proportion of individuals in an ongoing registry from each economic group can be compared to the proportion in the overall population in that economic group over time. The DHS generates a new Wealth Index for every new survey dataset it collects, which occurs about every five years for a given country. When a new DHS dataset becomes available, the proportion of households who fall into each cluster could be calculated. If the cluster sizes are roughly similar to their sizes in the previous survey, researchers could continue to use the old EconomicClusters model. If, however, some clusters had become so small as to no longer be meaningful as major economic groups in a given country, a new EconomicClusters model could be generated based on the new DHS dataset. If technology progressed to the point where an asset transitioned from being a luxury item to being outdated, then a new model could be generated for this reason as well. Registries or other longitudinal data collection instruments could then begin to use the new model. A specific EconomicClusters model can thus be updated over time to continue to generate economic models that are currently relevant for health disparities research.

Other limitations of all assets-based metrics include that they do not take into account other aspects of socioeconomic status such as education and occupation [5], which must be measured separately if they are variables of interest. Assets-based metrics also reflect wealth at the household level and to some extent at the community level for certain types of water and sanitation facilities, not at the individual level [4]. Because it is based on relatively few assets, the EconomicClusters metric should be interpreted as an approximation of an individual’s wealth and should be used only to determine general trends in health outcomes among populations with similar wealth, rather than to assign economic status to an individual for the purposes of providing services. Nonetheless, the EconomicClusters model fills a previously unmet need for an appropriate metric of economic status in studies where the goal is to assess a general level of material-based wealth when it is not possible to assess lengthier metrics.

## Conclusions

In summary, the EconomicClusters models for Cameroon and Ghana correlate consistently with three socioeconomic and health outcomes that are known to be associated with wealth, supporting the validity of the model as a metric of economic status for use in health disparities research. We have further optimized the utility of the model from its original version based on our collaborators’ feedback with early use of the model. The economic groups defined by the model can now be ordinally-ranked and combined if necessary based on their values of child HAZ, women’s literacy scores, and proportion of children who are deceased. In order to improve health equity as well as the overall health of populations, it is vital that researchers measure and address health disparities [1]. In time-constrained settings where consumption, expenditures, and Wealth Index are not feasible to assess, the EconomicClusters model could provide a standardized method of measuring health disparities that could be included in nearly any survey instrument. We hope that the EconomicClusters algorithm will provide a useful tool to facilitate health disparities research, policy, advocacy, and action in a feasible and sustainable manner around the world.
